# Health Tract, No. 17

**Published:** 1860-03

**Authors:** 


### HEALTH TRACT, No. 17.

---

### SABBATH PHYSIOLOGY.

The Almighty rested one seventh of the time of creation, commanding man to observe an equal repose. The neglect of this injunction will always, sooner or later, bring mental, moral, and physical death.

Rest is an invariable law of animal life. The busy heart beats, beats ever, from infancy to age, and yet for a large part of the time it is in a state of repose.

William Pitt died of apoplexy at the early age of forty-seven. When the destinies of nations hung in a large measure on his doings, he felt compelled to give an unremitting attention to affairs of state. Sabbath brought no rest to him, and soon the unwilling brain gave signs of exhaustion. But his presence in Parliament was conceived to be indispensable for explanation and defense of the public policy. Under such circumstances, it was his custom to eat heartily substantial food, most highly seasoned, just before going to his place, in order to afford the body that strength and to excite the mind to that activity deemed necessary to the momentous occasion. But under the high tension both brain and body perished prematurely.

Not long ago, one of the most active business men of England found his affairs so extended, that he deliberately determined to devote his Sabbaths to his accounts. He had a mind of a wide grasp. His views were so comprehensive, so far-seeing, that wealth came in upon him like a flood. He purchased a country seat at the cost of $400,000, determining that he would now have rest and quiet. But it was too late. As he stepped on his threshold after a survey of his late purchase, he became apoplectic. Although life was not destroyed, he only lives to be the wreck of a man.

It used to be said that a brick kiln " must be kept burning over the Sabbath ;" it is now known to be a fallacy. There can be no " must" against the divine command. Even now it is a received opinion that iron blast furnaces will bring ruin if not kept in continual operation. Eighteen years ago, an Englishman determined to keep the Sabbath holy as to them, with the result, as his books testified, that he made more iron in six days than he did before in seven ; that he made more iron in a given time, in proportion to the hands and number and size of the furnaces, than any establishment in England which was kept in operation during the Sabbath.

In our own New-York, the mind of a man who made half a million a year, went out in the night of madness and an early grave in only two years, from the very strain put upon it by a variety of enterprises, every one of which succeeded.

" It will take about five years to clear them off," said an observant master of an Ohio canal-boat, alluding to the wearing-out influences on the boatmen, who worked on Sabbaths as well as other days. As to the boatmen and firemen of the steamers on the Western rivers, which never lay by on the Sabbath, seven years is the average of life. The observance, therefore, of the seventh portion of our time for the purposes of rest is demonstrably a physiological necessity—a law of our nature.

